# Information and Communication Technology Use Among Low-Income Pregnant and Postpartum Women by Race and Ethnicity: A Cross-Sectional Study

**DOI:** 10.2196/jmir.3916

**Published:** 2015-07-03

**Authors:** Nymisha Chilukuri, Meredith West, Janice Lynn Henderson, Shari Lawson, Robert Ehsanipoor, Kathleen Costigan, Sarah Polk, Wendy Bennett

**Affiliations:** ^1^ Division of General Internal Medicine Johns Hopkins University School of Medicine Baltimore, MD United States; ^2^ Division of General Obstetrics and Gynecology Johns Hopkins University School of Medicine Baltimore, MD United States; ^3^ Division of General Pediatrics Johns Hopkins University School of Medicine Baltimore, MD United States

**Keywords:** pregnancy, health services accessibility, postpartum period, cell phones, text messaging, Internet, health status disparities, Hispanic Americans

## Abstract

**Background:**

Pregnancy and the postpartum period provide windows of opportunity to impact perinatal and lifelong preventive health behavior for women and their families, but these opportunities are often missed. Understanding racial/ethnic differences in information and communication technology (ICT) use could inform technology-based interventions in diverse populations.

**Objective:**

The objective of the study was to evaluate differences in the use of ICT between racial and ethnic groups as well as by English language proficiency.

**Methods:**

We conducted a cross-sectional study of 246 women who were aged 18 years or older and pregnant or within 1 year of delivery. They were recruited from 4 hospital-based outpatient clinics and completed a self-administered survey. We used multivariate regression analysis to evaluate the association between race/ethnicity and ICT (mobile phone/short message service [SMS] text message, Internet, and social network) usage by race/ethnicity and perceived English language proficiency after adjusting for age, income, marital status, and insurance status.

**Results:**

In all, 28% (69/246) of participants were Latina, 40% (98/246) were African American, 23% (56/246) were white, and 9% (23/246) from other racial/ethnic groups. Of the Latinas, 84% (58/69) reported limited English language proficiency and 59% (41/69) were uninsured. More than 90% of all participants reported mobile phone use, but more than 25% (65/246) had changed phone numbers 2 or more times in the past year. Compared to white women, African American women were less likely to SMS text message (OR 0.07, 95% CI 0.01-0.63) and Latinas were less likely to use the Internet to find others with similar concerns (OR 0.23, 95% CI 0.08-0.73). Women with limited English language proficiency were less likely to use the Internet overall (OR 0.30, 95% CI 0.09-0.99) or use email (OR 0.22, 95% CI 0.08-0.63) compared to women with adequate English language proficiency.

**Conclusions:**

Mobile phones are widely available for the delivery of health interventions to low-income, racially diverse pregnant and postpartum women, but disparities in Internet use and SMS text messaging exist. Interventions or programs requiring Web-based apps may have lower uptake unless alternatives are available, such as those adapted for limited English proficiency populations.

## Introduction

Information and communication technologies (ICT), including mobile phones (eg, smartphones and regular mobile phones), Internet, email, and social networking have the potential to improve care for underserved communities with reduced access to health care. Although pregnant women frequently utilize health care services, they are often lost to follow-up after delivery, even among those with high-risk pregnancies [1]. Prior studies in pregnant and postpartum women show increasing use of Internet for delivery of health information [2] and interest in mobile phone apps [3]. Potential participants in postpartum weight loss interventions report high interest in Web-based components [4]. ICT has the potential to engage with and educate women, thus promoting improved health for women and their families before and after delivery [5], but evidence to support the uptake among low-income racially and ethnically diverse populations is limited.

The Pew Research Center showed 90% of Americans used a mobile phone [6] and 87% reported at least some Internet use [7]. Among those who report Internet use, 91% send or read emails and 74% use social networking sites such as Facebook, Myspace, or LinkedIn. Those with higher education and in younger age groups report most frequent Internet use, suggesting that ICT may be an optimal tool for communication, monitoring, education, and even providing interventions to young women [7]. However, studies have shown that low-income populations have fewer available ICT resources, including a reliable home Internet connection, and also frequently change mobile phone numbers [8,9]. People with limited English proficiency face multiple barriers to health care and community services, indicating a high need for novel and effective outreach strategies to engage and communicate with this population [10-14].

To inform the development of ICT-based health-related programming to a diverse population of pregnant and postpartum women, we conducted a cross-sectional survey about their ICT usage. The aim of this study was to evaluate differences in the use of ICT between racial and ethnic groups and by English language proficiency. We hypothesized that pregnant and postpartum women frequently use ICT, including mobile phones, Internet, and social networking, and that rates of ICT use would be lowest among Latinas and those with limited English language proficiency. The rationale for the hypothesis was based on prior literature indicating that Latino adults, especially those with less education, had lower Internet usage [15].

We designed the study to inform the development of culturally appropriate ICT interventions to promote healthy lifestyle behaviors in the perinatal period.

## Methods

### Study Design

This was a cross-sectional study, using a one-time self-administered questionnaire, to describe ICT use among women who were pregnant or in the first year postpartum. The study was approved by the Johns Hopkins University institutional review board.

### Study Setting and Population

We recruited 246 women who were attending a clinical visit at 1 of 4 outpatient obstetric or pediatric clinical sites from 2 hospitals in Baltimore, MD, between January and April 2013. Three of the sites provided high-risk obstetric care. Women were eligible to participate if they were aged at least 18 years, reported that they could read English or Spanish either “well” or “very well” and were either pregnant or within 1 year of delivery.

### Survey Design and Data Collection

We designed a 68-item questionnaire with items adapted from validated instruments to assess sociodemographics, use of ICT, and self-efficacy (confidence on their ability) for accessing online health information. Self-efficacy was assessed using the question “How confident are you in your ability to find helpful and useful health information on the Internet?” adapted from the Perceived Efficacy in Patient-Physician Interactions (PEPPI) 5-item scale [16]. The stem of these items began with “How confident are you in your ability to...” which we adapted to be specific to health information on the Internet. The questions used a 5-point response scale (1=“not at all confident” and 5=“extremely confident”), which we dichotomized based on the distribution of responses into “extremely confident” and “somewhat confident” versus “neutral,” “not very,” and “not at all confident.” Questions on medical history and access to care were adapted from several national surveys including the Center for Disease Control and Prevention’s Behavioral Risk Factor Surveillance System [11] or the Pregnancy Risk Assessment Monitoring System Core and Standard Questionnaires [12]. ICT usage questions were adapted from The Pew Research Center’s questionnaires on Peer-to-Peer Healthcare [13] and Health Online [14]. The final survey was translated into Spanish. The English and Spanish versions of the survey were pilot-tested among English- and Spanish-speaking patients to ensure cultural relevance, understandability, readability (aiming for fifth grade reading level or less), and completion within 10-15 minutes. Eligible participants completed a 10-15 minute self-administered questionnaire either immediately before or after their outpatient clinic visit in a private space. We offered an audio-recorded version that read each question aloud using a CD player, but no one chose this version. Participants received a US $10 gift card.

### Measures

The primary outcome was use of ICT, which included use of mobile phone, short message service (SMS) text messaging, Internet, email, and social networks. We also asked whether participants used these technologies to identify health information for themselves and their families and, if so, what they found useful.

The main independent variables were self-reported race/ethnicity, coded as non-Hispanic black, non-Hispanic white, Hispanic (Latino), and other races, and self-perceived spoken English language proficiency. English language proficiency was assessed based on the US Census question and categorization, which had been incorporated into the Consumer Assessment of Healthcare Providers and Systems (CAHPS) Cultural Competence Supplemental Survey [17,18]: “How well do you speak English?” The response of “very well” was coded as “adequate” and any response of less than “very well” (“well,” “not well,” and “not at all”) was coded as having “limited English proficiency” based on previous validation of these cutpoints [17-19].

Other descriptors and covariates included sociodemographic variables [17] (eg, education level, income, marital status), pregnancy status, self-reported medical history, and health insurance.

### Data Analysis

We used descriptive statistics (*t* tests for continuous variables and chi-square tests for categorical variables) to assess and describe the characteristics of our sample and use of ICT by race/ethnicity. We created multivariate logistic regression models to assess the association between race and ethnicity and mode of ICT after adjusting for age and education level.

## Results

### Characteristics of Study Sample

The mean age in our sample of 246 women was 28 (SD 6) years with white women being slightly older (mean 31, SD 6 years) than Latina (mean 28, SD 6 years) and African American (mean 26, SD 6 years) women. Most women were pregnant at the time of the survey.In all, 28% (69/246) were Latina, 40% (98/246) were African American, 23% (56/246) were white, and 9% (23/246) were from other racial/ethnic groups, which included Asian (n=10), Native Hawaiian and Pacific Islander (n=1), American Indian/Alaskan Native (n=4), and multiethnic women (n=8). In all, 17% (12/69) of Latinas and 4% (33/98) of African Americans reported household incomes less than US $10,000 compared to 9% (5/56) of white women. For insurance status, 54% (132/246) were insured with Medicaid or Medicare, but 60% (41/69) of Latinas were uninsured and 36% (89/246) of women were employed either full or part time. For Latina women, 84% (58/69) reported limited spoken English language proficiency compared to 1.1% (2/177) of the other racial/ethnic groups. Latinas most commonly reported Mexico (29%, 20/69) and El Salvador (28%, 19/69) as countries of origin. The sample had a high prevalence of medical conditions including type 2 diabetes (7%, 16/246), gestational diabetes (11%, 28/246), hypertension (12%, 29/246), and overweight/obesity (56%, 138/246). For white and African American women, 84% (47/56) and 72% (71/98), respectively, reported having a primary care physician compared to 19% (13/69) of Latina women (Table 1).

**Table 1:** Characteristics of the sample of pregnant and postpartum women (N=246).

Characteristics		Total N=246	Latino n=69	African American n=98	White n=56	Other races n=23	*P*
Age (years), mean (SD)		28 (6)	28 (6)	26 (6)	31 (6)	29 (7)	<.001
Married or living with partner, n (%)		173 (70)	59 (86)	50 (51)	44 (79)	20 (87)	<.001
Currently pregnant, n (%)		206 (84)	53 (77)	85 (87)	50 (89)	18 (78)	.18
Limited English proficiency^a^		60 (24)	58 (84)	1 (1)	1 (2)	0 (0)	<.001
**Household income (US $),** ^c^ **n (%)**							<.001
	<$10,000	53 (22)	12 (17)	33 (34)	5 (9)	3 (13)	
	$10,000-$49,999	83 (34)	26 (38)	37 (38)	14 (25)	6 (26)	
	>$50,000	50 (20)	4 (6)	4 (4)	30 (54)	12 (52)	
**Education, n (%)**							<.001
	<Grade 12 or GED	60 (24)	36 (52)	13 (13)	7 (13)	4 (17)	
	Grade 12 or GED	83 (34)	22 (32)	48 (49)	10 (18)	3 (13)	
	>Grade 12 or GED	101 (41)	10 (14)	36 (37)	39 (70)	16 (70)	
**Insurance status, n (%)**							<.001
	Commercial plan	66 (27)	7 (10)	14 (14)	32 (57)	13 (57)	
	Medicaid/Medicare^c^	132 (54)	19 (28)	80 (82)	24 (43)	9 (39)	
	Uninsured	45 (18)	41 (59)	3 (3)	0 (0)	1 (4)	
**Employment status, n (%)**							.002
	Employed	89 (36)	15 (22)	35 (36)	27 (48)	12 (52)	
	Homemaker/maternity leave	68 (28)	33 (48)	16 (16)	14 (25)	5 (22)	
	Attending school	13 (5)	2 (3)	6 (6)	3 (5)	2 (9)	
	Unemployed	69 (28)	15 (22)	38 (39)	2 (4)	4 (17)	
**Medical history,** ^d^ **n (%)**							
	Type 2 diabetes	16 (7)	3 (4)	10 (10)	2 (4)	1 (4)	.30
	Gestational diabetes	28 (11)	7 (10)	8 (8)	12 (21)	1 (4)	.05
	High blood pressure	29 (12)	4 (6)	13 (13)	8 (14)	4 (17)	.31
	Overweight or obese^e^	138 (56)	30 (44)	70 (71)	27 (48)	11 (48)	.001
	Has primary care physician	147 (60)	13 (19)	71 (72)	47 (84)	16 (70)	<.001
**Phone and Internet use**							
	Uses mobile phone	234 (95)	65 (94)	90 (92)	56 (100)	23 (100)	.15
	Uses smartphone	172 (74)	38 (55)	69 (77)	47 (84)	18 (78)	.004
	≥2 mobile phone numbers in last 12 months	65 (26)	17 (25)	34 (35)	10 (18)	4 (17)	.08
	Has home phone	106 (43)	25 (36)	48 (49)	24 (43)	9 (39)	.69
	Uses Internet	209 (85)	43 (62)	90 (92)	54 (96)	22 (96)	<.001
	High (vs low) self-efficacy for using Internet	145 (59)	20 (29)	62 (63)	44 (79)	19 (83)	<.001
**ICT outcomes**							
	SMS text messaging	222 (90)	61 (88)	83 (85)	55 (98)	23 (100)	.02
	Email	193 (79)	35 (51)	84 (86)	52 (93)	22 (96)	<.001
	Internet	209 (85)	43 (62)	90 (92)	54 (96)	22 (96)	<.001
	Use of Internet to find health info	182 (74)	35 (51)	77 (79)	49 (88)	21 (91)	<.001
	Using Internet to find others with similar concerns	102 (42)	8 (12)	48 (49)	36 (64)	10 (43)	<.001
	Social networking	187 (76)	37 (54)	81 (83)	49 (88)	20 (87)	<.001

^a^ Limited language proficiency defined less than “very well” on the question “How well do you speak English?”

^b^ Survey item provided the option of declining to disclose income. A total of 24% (60/246) declined: 39% (27/69) Latino, 25% (24/98) African American, 13% (7/56) white, 9% (7/23) other races.

^c^ Proportion with Medicaid (vs Medicare) was 52% (128/246) with 26% (18/69) Latinas, 77% (77/98) African American, 43% (24/56) white, and 39% (9/23) with other races.

^d^ Medical history is self-reported.

^e^ Body mass index (BMI) calculated using self-reported prepregnancy weight (kg) divided by the square of self-reported height (m^2^). Overweight or obese defined as BMI ≥30 kg/m^2^.

### Rates of Information and Communication Technology Usage

**Figure 1:**
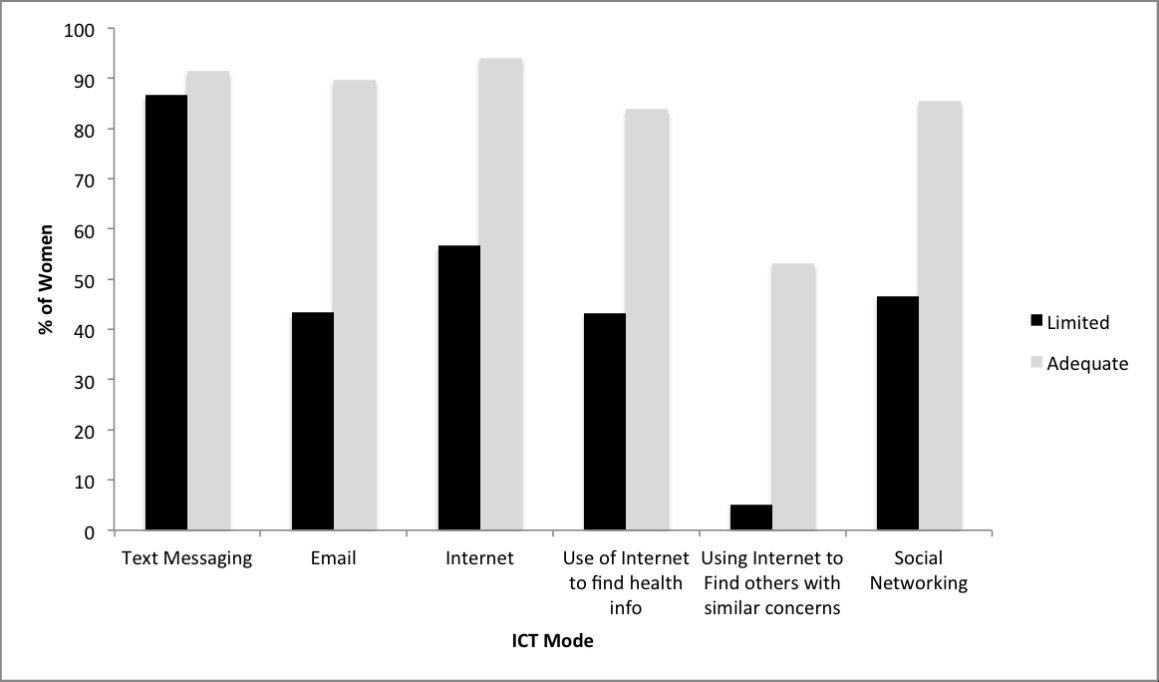
Rates of use of information and communication technology modality and function by English language proficiency (adequate vs limited).

Mobile phone use was greater than 90% (234/246) among all racial and ethnic groups with African American women reporting the lowest rate of 92% (90/98) (Table 1). Compared with African American and white women, fewer Latina women used smartphones (55%, 38/69), social networking sites (54%, 37/69), or accessed the Internet (62%, 43/69) (Table 1). However, the majority of women in all racial/ethnic groups used mobile phones for SMS text messaging, although the rate was slightly lower for African American women (Latina: 88%, 61/69; African American: 85%, 83/98; white: 98%, 55/56) (Table 1). More than one-quarter of the sample (26%, 65/246) reported having 2 or more different mobile phone numbers in the past 12 months and 43% (106/246) of women reported having a home phone number or landline. Compared with women with adequate spoken English language proficiency, women with limited English language proficiency less frequently used all forms of ICT (Figure 1). Among Latinas, those with limited English proficiency had lower use of Internet (38/69, 55%) compared to Latinas with adequate English language proficiency (55%, 32/58 vs 100%, 11/11, *P*=.005) (not shown).

### Odds of Information and Communication Technology Use by Race/Ethnicity and English Language Proficiency

Compared to a white reference group, African American women were statistically significantly less likely to report SMS text messaging (OR 0.08, 95% CI 0.01-0.67) after adjustment for age and education (Table 2). Compared to white women, Latinas were less likely to report using the Internet (OR 0.15, 95% CI 0.03-0.78), email (OR 0.17, 95% CI 0.05-0.61), social networking (OR 0.27, 95% CI 0.09-0.75), Internet use to find other people with similar health concerns (OR 0.16, 95% CI 0.06-0.43), and had lower self-efficacy for health information–related Internet use (OR 0.26, 95% CI 0.10-0.68) (Table 2).

**Table 2:** Odds of using information and communication technology (ICT) by race/ethnicity.^a^

ICT use	Latino, OR (95% CI) n=69	African American, OR (95% CI) n=98
SMS text message	0.18 (0.02-1.61)	0.08 (0.01-0.67)
Internet use^b^	0.15 (0.03-0.78)	0.54 (0.10-3.04)
Email use	0.17 (0.05-0.61)	0.52 (0.14-1.91)
Social networking	0.27 (0.09-0.75)	0.58 (0.20-1.66)
Internet used to obtain health Information^b^	0.38 (0.14-1.07)	0.90 (0.32-2.54)
Internet used to find others with similar concerns	0.16 (0.06-0.43)	0.86 (0.39-1.89)
High (vs low) self-efficacy for Internet use^c^	0.26 (0.10-0.68)	0.62 (0.26-1.49)

^a^ Reference=white race. Model adjusted for age and education.

^b^ Includes accessing Internet via mobile phone or computer.

^c^ Self-efficacy assessed using question “How confident are you in your ability to find helpful and useful health information on the Internet?” and categorized as high=extremely confident and somewhat confident vs low=neutral, not very, and not at all confident.

Compared to women with adequate English language proficiency, women with lower English language proficiency were equally likely to SMS text message (OR 0.97, 95% CI 0.34-2.72), but had a lower likelihood of using the Internet (OR 0.20, 95% CI 0.08-0.47), email (OR 0.19, 95% CI 0.09-0.41), social networking (OR 0.27, 95% CI 0.13-0.57), Internet used to obtain health information (OR 0.27, 95% CI 0.13-0.56), and Internet used to find others with similar concerns (OR 0.08, 95% CI 0.02-0.28) (Table 3).

**Table 3:** Odds of information and communication technology (ICT) use for women with low vs adequate English proficiency.^a^

ICT use	Low English language proficiency, OR (95% CI)
SMS text message	0.97 (0.34-2.72)
Internet use^b^	0.20 (0.08-0.47)
Email use	0.19 (0.09-0.41)
Social networking	0.27 (0.13-0.57)
Internet used to obtain health information^b^	0.27 (0.13-0.56)
Internet used to find others with similar concerns	0.08 (0.02-0.28)
High (vs low) self-efficacy for Internet use^c^	0.21 (0.09-0.45)

^a^ Model adjusted for age and education.

^b^ Includes accessing Internet via mobile phone or computer.

^c^ Self-efficacy assessed using question “How confident are you in your ability to find helpful and useful health information on the Internet?” and categorized as high=extremely confident and somewhat confident vs low=neutral, not very, and not at all confident.

## Discussion

In this sample of low-income, racially and ethnically diverse pregnant and postpartum women, mobile phone and SMS text message usage were common across all racial/ethnic groups. Although more than 85% of all participants reported SMS text messaging, African American women were less likely to text compared to white women. The rates of mobile phone usage reported in our survey were similar to The Pew Research Center’s Internet Project Survey data showing 90% of people own cell phones, including 90% of whites and African Americans and 92% of Hispanics [6]. Additionally, we confirmed low rates of landline use (57% did not own landlines) consistent with Pew Internet Research findings (41%). In the Pew survey, households with lower socioeconomic status and Hispanics had higher rates of not having landlines (56.2% and 53.1%, respectively) [20]. Although our sample generally reported high mobile phone and SMS text messaging rates, we identified disparities in Internet, email, and social networking use by racial/ethnic groups and limited English language proficiency. Our results suggest that mobile phones are potentially useful modalities for the delivery of health interventions to low-income pregnant and postpartum women, but interventions requiring Web-based apps may have lower uptake unless alternatives (ie, paper) and Spanish translations are available.

Other studies have also reported lower rates of Internet usage among Latinas, but without close examination of the role of English language proficiency. A large cross-sectional survey of 3181 young women attending reproductive health clinics in Texas reported 92.7% of whites and 92.9% of African American women, but only 67.5% of Latinos, used the Internet. Hispanic women reported barriers to Internet use including cost, not having a computer at home, and not knowing how to use a computer [21]. However, the impact of English language proficiency on differential ICT use was not described. Notably, more than 80% of Latinas in our study had limited English language proficiency and 17% spoke any English, which is in contrast to the Latino population sampled in the Pew Internet Research Survey in which 65% were English speaking (either English dominant or bilingual) and proficiency was not assessed. The large difference in English proficiency likely accounts for the disparity we noted in Internet use, in which only 62% of Latinas in our sample used the Internet compared to 78% of Latinos in the Pew Survey [22]. In addition, more than half of the Latinas in our sample reported less than a high school education; from other surveys from this community, more than one-third likely have less than a sixth grade education [15] indicating lower literacy, including Spanish literacy. Our results suggest that English language and literacy are major barriers for women to use and access the Internet. The development of technology-based interventions, especially those that require Internet components, should be translated into Spanish, designed for people with lower literacy, and culturally adapted for Latinos to have the greatest potential impact.

Despite these disparities in Internet usage, our study supports mobile phone-based interventions in a low-income, racially diverse population. Growing evidence supports mobile phone-based interventions to impact health behaviors, but few studies have focused on pregnancy and postpartum health [23]. One example of a large-scale SMS text messaging program aimed at improving prenatal care and pregnancy outcomes is the Text4Baby program launched in 2010 by the Centers for Disease Control and Prevention. Women who sign up receive texts containing information about prenatal and postpartum health behaviors and services [24]. However, a randomized controlled trial of 123 women (approximately 80% of whom were Spanish speaking and 75.6% who had participated in the Special Supplemental Nutrition Program for Women, Infants, and Children [WIC] program) did not show a difference between text4baby intervention and usual care control in terms of changes in self-reported health behaviors, but no birth or utilization of care outcomes were reported [25]. Adaptation of the SMS text messaging programs for low-income women is especially important. The WIC program serves low-income women and children and has also been focused on improving care delivery through mobile-based apps [25,26]. A WIC program in Atlanta, GA, tested the text4baby program to assess participants’ enrollment and satisfaction in 468 (91% African American) participants. Only 51% of women provided with enrollment instructions attempted to enroll in the program; among these, 69% successfully enrolled mostly via SMS text message (vs online). Higher education and higher incomes were associated with increased enrollment, indicating that the enrollment process may have more barriers for less educated and poorer women [27]. This study notes the importance of testing actual use of ICT interventions in low-income populations to reduce their risk of widening the disparities that they were designed to address.

An additional challenge to implementing and sustaining SMS text messaging interventions and programs is the frequency with which women reported changing mobile phones or phone numbers. In our sample, more than one-quarter of women reported having 2 or more cell phone numbers in the last 12 months, with the highest rates among African American women (35%) and Latinas (25%). To facilitate intervention adherence involving use of mobile phones, studies have budgeted funding to provide mobile phone minutes or plans or even phones to participate to enhance participation rates, but this may not be cost-effective for community-based programs and the aforementioned intervention was not offered in multiple languages [28]. Other studies have required an unlimited short messaging plan [29], but this may exclude lower income populations [30].

The major strengths of our study are including both obstetric and pediatric sites, and identifying a higher risk population of women with medical comorbidities and a racially and ethnically diverse sample. We also collected information about perceived English language proficiency to analyze the results not only by racial and ethnic groups, but also by English language proficiency.

There are several limitations of our study. First, we surveyed a convenience sample of women who were attending one of several clinical sites and may have missed women who did not receive prenatal care or who did not attend visits. This may have made the study’s results less generalizable to other women in Baltimore, but because we collected data over several months, women had multiple opportunities to attend visits and complete the surveys. Second, because this was a self-administered questionnaire, we screened out 5 participants who had self-reported low literacy in English or Spanish as part of study eligibility and thus our results provide a “best-case scenario” for Internet and mobile phone use among literate women. Third, the cross-sectional design limits our ability to assess causality. Fourth, our study examined racial and ethnic differences in Internet and mobile phone usage in a population of low-income women in Baltimore, MD, and these differences may be different in other cities in the United States. Fifth, there is a potential that results being attributed to ethnicity may in fact be due to income instead, especially because the latter was not included in the multivariate regression analysis. Sixth, our study did not account for potential differences in ICT usage for nulliparous versus multiparous women because more experienced mothers may differ in their level of need for information.

In conclusion, our findings show that the racial and ethnic digital divide regarding mobile phone use and SMS text messaging is diminishing, but persists for Internet, email, and social networking by race and ethnicity and particularly for women with limited English language proficiency. These findings support developing linguistically and culturally appropriate mobile phone and SMS text messaging interventions for women of all ethnicities and language proficiencies to promote improved healthy lifestyle behaviors, specifically in the perinatal period.

## Abbreviations

BMI: body mass index
CAHPS: Consumer Assessment of Healthcare Providers and Systems
ICT: information and communication technology
PEPPI: Perceived Efficacy in Patient-Physician Interactions
SMS: short message service
WIC: Special Supplemental Nutrition Program for Women, Infants, and Children
